# Correction: Numerical investigation of LDL nanoparticle collision in coronary artery grafts with porous wall and different implantation angles and two state of inlet velocity

**DOI:** 10.1371/journal.pone.0305829

**Published:** 2024-06-14

**Authors:** The PLOS ONE Staff

In the Surfaces and parameter subsection of Results, the equation is incorrect. A vector in the letter N is placed in error. Please see the correct equation here.


$$Normalized\;Collied\;Particles\to NCP=10000*\left(\frac{number\;of\;nanoparticles\;colliding\;the\;surface}{total\;number\;of\;injected\;nanoparticles}\right)$$

The corresponding author’s email address is incorrect. Reza Karimian’s email address is: r.karimian@alumni.iut.ac.ir.

The publisher apologizes for the errors.
